# Misinformation and Junk Science in Obstetrics Medical Malpractice: Newborn Brain Injury

**DOI:** 10.1097/og9.0000000000000208

**Published:** 2026-09-22

**Authors:** Michael G. Ross

**Affiliations:** Department of Obstetrics and Gynecology and Public Health, Geffen School of Medicine, and Fielding School of Public Health, UCLA, Los Angeles, California; and Institute for Women's and Children's Health, The Lundquist Institute, Los Angeles, California.

## Abstract

Medical–legal liability remains a constant concern to obstetricians because the majority of physicians are sued at least once in their career. The emotional and psychologic stress may disrupt both clinical practice and personal lives. A physician defendant may be able to address liability allegations but may be more challenged by causation opinions that are not based on a scientific foundation. Junk science or misinformation may be promulgated by either plaintiff or defendant representatives, with judges empowered to determine the scientific validity of claims (ie, Daubert, Frye hearings). Because cerebral palsy cases are among the most common and highest liability cost, misinformation theories are often proposed in support of an allegation. Herein are presented the basic science critiques of several examples of misinformation and junk science theories that allege to explain causes of fetal and newborn brain injury. Enhanced basic science and human physiology education during obstetrics and gynecology residency training may both aid in the clinical management of atypical, challenging cases and provide the knowledge base to allow physicians to confidently address junk science allegations should they arise.

## Abstract 2

Obstetric malpractice allegations of fetal and newborn brain injury must be based on established scientific evidence of causation.

The American Medical Association 2026 report demonstrated that medical liability premiums rose for the seventh consecutive year. Premiums for obstetrics and gynecology are among the highest among all specialties, with annual rates exceeding $200,000 in several states.^1^ From 2022 to 2024, obstetrics and gynecology represented the highest proportion of physicians ever sued in their career (59.6%), with 4.6% of obstetrics and gynecology physicians sued in the prior year. As previously discussed,^2^ the consequences of malpractice claims may have adverse effects on physician practice in that medical–legal concerns can affect clinical management, resulting in excessive and often unnecessary interventions (eg, cesarean deliveries) and excessive health care costs. It is important to note that being named as a defendant also may result in marked psychological and emotional stress.^3^

Among paid malpractice claims in the United States (2005–2009), nearly 97% were settled outside court, and only 3% were judged in court.^4^ Despite the lack of trial, settlements typically include deposition testimony from treating parties and both plaintiff and defense experts. A strong plaintiff expert can increase settlement value by valid claims of liability and causation, whereas a strong defense expert can weaken the claim, give defense counsel leverage, or even support motions to exclude select plaintiff expert testimony.^5^

“Junk science” in malpractice litigation refers to expert opinions or medical theories that sound scientific but lack reliable methodology, sufficient foundation, or general acceptance under the applicable admissibility standard. The validity of scientific explanations may be challenged by either the plaintiff or the defense under Frye or Daubert standards. Frye, from a 1923 case (*Frye v United States*), uses a “general acceptance” test: Expert methods must be widely accepted in the relevant scientific community to be allowed as evidence. The more commonly used Daubert, set by the 1993 Supreme Court case (*Daubert v Merrell Dow*), replaced Frye federally and requires judges to act as “gatekeepers” assessing whether testimony is reliable and relevant using multiple reliability factors, including peer review and testability, as well as general acceptance. Judges face significant challenges when making Frye and Daubert decisions because they must evaluate complex and often discordant scientific evidence, often without trained expertise. Whereas conflicting expert testimony in civil cases is nearly universal, judges are empowered as gatekeepers to discern the scientific reliability of expert theories. Approximately half of judges report that their formal education had not prepared them to deal with scientific evidence, even though most (91%) still feel confident in their gatekeeping role.^6^ This mismatch may be viewed as either a positive or negative in adjudicating the admissibility of testimony.

As a result of the comparatively high judgments in obstetric cases,^7^ diametrically opposing expert opinions are often presented. A previous report^2^ discussed topics of junk science or misinformation, including “fetal reserve,” uterine tachysystole, maternal versus clinician forces in alleviation of shoulder dystocia, and intrauterine pressure catheter measurement of uterine tone. In the present report, topics of controversy in causation of fetal and newborn brain injury are addressed, with clarification of the scientific basis.

## DOES VACUUM-INDUCED INFANT SUBGALEAL BLEED INDICATE PHYSICIAN NEGLIGENCE?

Vacuum-assisted vaginal deliveries constitute approximately 3.3% of deliveries undergoing trial of labor.^8^ It was long stated that vacuum devices were specifically designed to pop off the fetal scalp before causing infant harm,^9^ and they were generally considered to have a very high safety profile.^10^ Consequently, an adverse event from vacuum delivery might be assumed to indicate physician negligence. However, the U.S. Food and Drug Administration (FDA) issued a public health advisory on May 21, 1998, to advise physicians and health care practitioners that vacuum-assisted delivery devices may cause serious or fatal complications, including subgaleal hematoma and intracranial hemorrhage. The FDA attributed part, although not all, of the increases to the marked increase in the use of vacuum-assisted delivery devices. After the FDA alert, there was a 22-fold increase in the rate of reported adverse events associated with vacuum delivery,^11^ suggesting previous underreporting.

Mechanically, vacuum extractors exert traction only on the scalp skin. The “vacuum pressure” is not transmitted inside the skull, even when the cup is properly placed over the posterior fontanelle. Anatomically, emissary veins pass from the dural venous sinuses through the subgaleal space to the scalp. Stretching the scalp may tear these veins, resulting in a subgaleal bleed. Thus, it is vacuum traction on the scalp, not vacuum detachments or pop-offs, that may induce injury. The diffuse spread of a subgaleal hematoma across suture lines, combined with head molding, caput, and vacuum-associated chignon, makes it extremely difficult to diagnose the bleed. The actual bleed (and blood volume loss) generally begins before head delivery, coinciding with the vacuum use, and continues postnatally. Thus, an initial infant hematocrit before newborn fluid administration and volume equilibration may not indicate anemia, although pallor and hypotension may reflect hypovolemia.

The subgaleal space can accumulate a massive volume of blood (approximately 250 mL in infants). Because the blood volume of a newborn approximates only 80 mL/kg, the infant may rapidly lose 30–50% of blood volume before recognition, resulting in severe morbidity or mortality. With an incidence of symptomatic subgaleal bleeds of 1.5–5.5 per 10,000 births,^12,13^ many obstetricians may fortunately never experience a case. To facilitate early recognition, neonatal teams should avoid covering the newborn head with beanie caps while maintaining vigilance in the immediate postnatal care of infants delivered by vacuum assistance. Beyond neonatal volume replacement, there are no demonstrated effective treatments.

It is important to note that there are no known factors associated with vacuum delivery that modify the subgaleal risk; a subgaleal bleed may be precipitated with the first or the last vacuum traction. Thus, the American College of Obstetricians and Gynecologists (ACOG) has stated, “There are no adequate data to generate an evidence-based guideline for the number of forceps pulls or vacuum detachments that should be allowed … .”^14^ Nevertheless, there is evidence that excessive vacuum duration and the number of pulls and pop-offs may be associated with scalp injury^15^ or intracranial hemorrhage^16^ other than subgaleal bleed. Thus, the injury of a vacuum-associated subgaleal bleed is generally a risk of the nature of vacuum delivery and not de facto indicative of negligence or improper physician technique.

The original vacuum cup (ie, Malmstrom) was stainless steel with a chain handle. Modern vacuum extractor cups are made of silicone rubber, polyurethane, or thermoplastic elastomer and are bell or mushroom shaped with a variety of sizes, edges, and handles. With each new FDA-approved vacuum, only a 510K claim of “substantial equivalence” to other vacuum cups is required, with no required testing of actual traction force before detachment. Paradoxically, improved traction may ultimately result in increased scalp stretching and a higher frequency of subgaleal bleeds. Only an FDA and industry effort to assess and moderate actual traction force before detachment can potentially improve the safety of vacuum extractors.

## DOES FETAL HEAD COMPRESSION RESULT IN LOCAL BRAIN ISCHEMIA?

The fetal head is subjected to pressure related to both uterine contractions and maternal pushing during labor. In obstetric malpractice cases, fetal head compression has been alleged to lead to profound cerebral ischemia and brain injury resulting from increased intracranial pressure.^17^ It has been asserted that cerebral compression results from excessive uterine activity, as evidenced by multiple associations that may ultimately encompass a majority of laboring patients (Box 1).

Box 1. **Allegation of Excessive Uterine Activity for Cerebral Compression Injury**

Prolonged latent phase, active phase, second stage

Uterine hyperstimulation or tachysystole

- Excessive contraction frequency, duration, strength, or basal tone
- Reduced uterine “resting time”
- Excessive dose or duration of oxytocin, independently of contraction response

Cephalopelvic disproportion, as documented in an operative report

“Excessive” molding as documented in newborn assessment

Malposition as occiput posterior

“Difficult delivery”

The alleged increased force on the fetal head may be a consequence of intrauterine pressure or force against the body pelvis. Uterine contraction-induced intrauterine pressure increase^18^ is transmitted throughout the fetal head and body, much as scuba diving increases ambient pressure without affecting cerebral blood flow. Thus, increased amniotic pressure does not cause a true increase in intracranial pressure.^18^ Alternatively, pressure on the fetal skull against the bony pelvis occurs with contractions and maternal pushing. To adapt to the markedly larger fetal head size compared with subhuman primates,^19,20^ evolution led to unfused cranial sutures to permit head molding in response to the limited pelvic size. Thus, fetal head compression and the accompanying head molding are an intrinsic component of term labor.^21^

As Heyborne^22^ concluded, despite the increase in extracranial pressure on the fetal skull with uterine contractions and pushing, data do not support a significant increase in intracranial pressure, a reduction in cerebral circulation or oxygenation, or an change in cerebral function. One may view the effect of external pressure on internal pressure similar to the pressure on an automobile tire. With an automobile raised on a lift, a normal internal tire (intracranial) pressure approximates 35 lb/sq in. Lowering the approximately 4,000-lb. car to the ground dramatically increases the external (extracranial) pressure, but the internal pressure itself remains at 35 lb/sq in. This physics property differs from an artificial pressure originating from inside the cranium (eg, volume infusions into the lateral ventricles of fetal lambs).^23^ However, even under these conditions, the near-term fetus protects cerebral blood by vasoconstriction and increased systemic blood pressure (Cushing reflex), as well as increased fractional oxygen extraction.

Despite this evidence, plaintiffs have alleged that fetal cerebral compression results in localized cerebral ischemia and the development of brain injury, in the absence of systemic fetal acidosis. Evidence indicates that approximately 70–80% of cerebral palsy cases are associated with antenatal factors.^24–27^ Infants may recover from a prenatal, acute hypoxic episode and present with preexisting neurologic damage despite normal umbilical acid–base and blood gases. To postulate that intrapartum fetal head compression results in select cerebral hypoxia but not systemic acidosis is an example of scientific misinformation or junk science.

## DO EARLY NEWBORN INCREASED NUCLEATED RED BLOOD CELLS INDICATE A PREEXISTING HYPOXIC INSULT?

Small numbers of nucleated red blood cells (RBCs) are commonly found in the blood of normal newborns immediately after birth,^28^ whereas increased nucleated RBCs may occur in response to fetal anemia, blood loss, and significant hypoxia. The timing and degree of elevation of newborn nucleated RBCs have been controversial, with claims that the time required to elevate blood nucleated RBCs indicates that the infant must have had a hypoxic–ischemic event predating labor.

The time course of nucleated RBC count has focused on an erythropoietin-induced mechanism. Sodium nitrite–induced hypoxia in fetal sheep increased plasma erythropoietin at 4–5 hours,^29^ whereas infusing synthetic erythropoietin into human newborns increased circulating nucleated RBCs 24–36 hours later.^30^ These results suggested that hypoxia-induced release of nucleated RBCs is mediated by erythropoietin and requires more than 24 hours for increased circulating nucleated RBCs.

However, multiple reports have confirmed that nucleated RBCs may be rapidly released from hematopoietic tissues after a hypoxic episode. Small case reports confirm increased nucleated RBCs in infant blood within 20 minutes to 6 hours of acute hypoxia.^31,32^ Korst et al^33^ reported that infants with a reactive fetal heart rate on admission followed by an acute prolonged heart rate deceleration had significantly increased newborn nucleated RBC count (12.6±13.4 versus 3.4±3.0 nuclear RBCs/100 white blood cells [WBCs]). Similarly, nucleated RBC counts of infants born after acute uterine rupture were significantly increased (10.3±6.7 versus 3.4±3.0 nucleated RBCs/100 WBCs).^34^ These findings indicate that hypoxia-induced acute nucleated RBC release is likely secondary to systemic factors other than erythropoietin.

Together, these reports and others suggest that nucleated RBC counts of 10–20/100 WBCs may represent a relatively rapid response to acute hypoxia. In contrast, Hankins et al reported that 41% of infants having a sentinel hypoxic event occurring during or immediately preceding labor demonstrate nucleated RBC count ≥26/100 WBCs within 5 days of life.^35^ Thus, within obstetric malpractice litigation, proposals that a modest increase in nucleated RBCs (eg, 5–15/100 WBCs) rules out an intrapartum sentinel hypoxic event are not based on scientific data.

## DOES LACTATE WASHOUT INDICATE PREDELIVERY SEVERE ACIDOSIS?

Lactate washout may be defined as the rapid clearing of accumulated lactate from poorly perfused tissue or blood after circulation is restored, such that lactate levels rise after resuscitation or reperfusion. A marked increase in lactate or base deficit from an umbilical blood sample to an early newborn blood sample has been claimed to be caused by lactate washout after reperfusion of a newborn after a predelivery fetal hypoxic event. However, this claim may misrepresent acid–base physiology.

Lactate, a product of anaerobic metabolism of pyruvate by lactate dehydrogenase, is cleared out of the cell along with hydrogen ions by specific proteins (monocarboxylate transporters), with the flux rate dependent on the diffusion gradient.^36^ Under conditions of complete cord occlusion, the fetus typically maintains a ventricular pacemaker-mediated cardiac rate (50–70 bpm). Thus, the proximal umbilical artery continues pulsation with blood mixing. With relief of the cord obstruction at the moment of birth, the umbilical artery lactate and base deficit commonly reflect the newborn acid–base status at birth. However, even with implementation of universal umbilical cord blood pH analysis, only a single umbilical vessel may be sampled (20–60%).^37^ The larger-caliber umbilical vein is generally easier to sample, resulting in the single vessel often thought to represent the infant's acid–base status at birth. When a later newborn blood sample reveals a marked increase in acidosis, claims of lactate washout have been proposed.

There are two physiologic explanations for these findings. Because umbilical venous flow is absent during total cord occlusion, the umbilical vein base deficit represents the fetal status immediately before the cord occlusion. During fetal bradycardia from a complete cord occlusion, fetal base deficit increases at approximately 1 mmol/L per 2 minutes.^38^ A 10-minute bradycardia would result in an umbilical artery base deficit approximately 5 mmol/L greater than the umbilical vein. If only the vein is measured at birth, a later newborn blood sample may falsely appear to represent lactate washout. Alternatively, if both vessels are measured at birth (and confirmed to be both artery and vein), a marked increase in base deficit in the early newborn period is most often a consequence of a difficult neonatal resuscitation (eg, delayed neonatal transition, cardiovascular anomalies, impaired ventilation [eg, meconium aspiration, esophageal intubation], sepsis, hypovolemia, or anemia). With newborn cardiopulmonary resuscitation and cardiac compression, carotid blood flow and blood pressure are only a fraction of normal.^39^ With the increased newborn oxygen demands, cardiopulmonary resuscitation results in a base deficit increase of approximately 0.5 mmol/L per minute. Thus, a prolonged newborn resuscitation may result in a significant increase in base deficit from measures at the time of birth.

Consequently, a marked increase in lactate and base deficit from birth to a newborn blood sample may indicate that only the umbilical vein was sampled at delivery or, alternatively, may reflect a prolonged and difficult neonatal resuscitation. Together, these findings suggest that lactate washout represents an uncommon cause of newborn acidosis.

## DISCUSSION

Newborn encephalopathy and cerebral palsy are among the most common and highest liability issues in obstetric malpractice. The topics addressed here (subgaleal bleed, head compression, lactate washout, and nuclear RBC counts) have relevance to infant brain injury. Whereas physician liability for subgaleal bleeds and head compression–induced fetal brain injury is more often allegations of plaintiff positions, theories of lactate washout and implications of nucleated RBC counts may be alleged by and potentially misinterpreted by both defendants and plaintiffs. The review of the scientific basis of each of these topics is not meant to address standard of care or physician liability but rather to ensure that causation allegations or explanations are credible.

Expert witnesses bear an ethical responsibility to avoid bias and to emphasize “expert testimony” rather than “expert opinion.” The ACOG Committee Opinion 374 “Expert Testimony”^40^ emphasizes the role of the expert witness, with a focus on distinguishing between medical malpractice and medical maloccurrence. Whereas standard-of-care opinions can often be validated with society guidelines (eg, ACOG, Society for Maternal-Fetal Medicine), causation opinions may rely on a knowledge of maternal or fetal physiology, pharmacology, clinical obstetrics, and perhaps basic chemistry, math, or physics. Importantly, the ACOG Expert Witness Affirmation includes the statement “I will make every effort to determine whether there is a causal relationship between the alleged substandard practice and the medical outcome.” Thus, it is incumbent on the expert witness to present scientifically valid causation opinions.

Adverse events in obstetrics and gynecology have often been attributed to delay of care, coordination of care, and scarcity or supply of personnel.^41^ In an effort to address these issues, multiple obstetric patient safety programs have been proposed or implemented,^42,43^ with demonstration of promising results.^42,44^ These programs have often emphasized standardized clinical protocols, communication processes, specialist consults, team drills, and data safety huddles. However, a recent risk-prevention review found 64% of cases included issues of clinical judgment or inadequate patient assessment, 47% included technical errors, and 40% involved decision making or timing of therapy. Less than 20% of obstetrics liability cases involved communication breakdown with the care team.^45^ Clearly, these factors highlight the need for a continued focus on education during residency training and continuation of lifelong learning.

It has long been recognized that biomedical research is limited in obstetrics and gynecology departments compared with other clinical departments. Obstetrics and gynecology departments receive less than 1% of total NIH funding across all medical school departments for early-stage investigators,^46^ and only a small subset of academic obstetrics and gynecology programs have strong basic science programs. Because the majority of obstetrics and gynecology programs are community based,^47^ it is not surprising that basic science education is limited during obstetrics and gynecology residencies. Although it is difficult to prove the absolute value of basic science in clinical practice, evidence indicates that improved obstetric care and avoidance of substandard care optimize patient outcomes and reduce liability.^48^ Basic science knowledge improves clinical decision making when it is cognitively integrated with clinical features during learning, allowing a clinician to later recognize a case as an instance of an underlying mechanism^49–51^ and to facilitate diagnostic reasoning and clinical management of complex cases.^52^ Therefore, Council on Resident Education in Obstetrics and Gynecology Educational Objectives embed “Basic Science/Mechanisms of Disease” as its own unit within the obstetrics curriculum.

An enhanced knowledge of basic science and human physiology, integrated with practicing clinical skills and society guidelines, may reduce adverse outcomes in atypical, challenging cases. Furthermore, this knowledge base may allow physicians to confidently address misinformation and junk science allegations should they arise. With the increasing use of society guidelines, care algorithms, and artificial intelligence–derived decision making, it is essential that training programs enhance basic science education into obstetrics and gynecology curriculums.
